# Supplementary material for: ATP8B1 Knockdown Activated the Choline Metabolism Pathway and Induced High-Level Intracellular REDOX Homeostasis in Lung Squamous Cell Carcinoma
Source: Cancers (Basel). 2022 Feb 7;14(3):835. doi: 10.3390/cancers14030835 (PMC8834475; doi:10.3390/cancers14030835)
Supplement: Supplementary file 1 [file cancers-14-00835-s001.zip › cancers-1512918-supplementary.pdf]

cancers MDPI

Supplementary Materials: ATP8B1 Knockdown Activated the Choline Metabolism Pathway and Induced High−Level Intracellular REDOX Homeostasis in Lung Squamous Cell Carcinoma

Xiao Zhang, Rui Zhang, Pengpeng Liu, Runjiao Zhang, Junya Ning, Yingnan Ye, Wenwen Yu and Jinpu Yu

Table S1. The sequences of primers for detection.

No.	Gene Name	Forward Primer	Reverse Primer
1	ATP8B1	5′-ATGATGGAGAAGACGATACACC-3′	5′-CAGCATAGTACATTTGCAGGTC-3′
2	CHKA	5′-GCCTATCTGTGGTGCAAGGAGTTC-3′	5′-GCCGCCTCTGATGACACTGATG-3′
3	L1−SMYD3	5′-TTTAAGCCGGTCCGAAAAGC-3′	5′-TCTGCCATGTGTCACTCTCA-3′
4	L1−CBWD2	5′-TTTAAGCCCGTCGGAAAAGC-3′	5′-TGACCTCCATGCAGTGATTG-3′
5	L1−FGGY	5′-CGCCTTGCAGTTTGATCTCA-3′	5′-TGTGGCCAGGTAGAGAATGG-3′
6	L1−LOC105369887	5′-GCAATCAGCGAGACTCCATG-3′	5′-GCTGTGATGGGCTTCTCATT-3′
7	L1−GBP1P1	5′-GAAAAGCGCAGTATTCGGGT-3′	5′-GGCGAAGATCCAGGAGTCAT-3′
8	L1−ACVR1C	5′-GAGTGACCCGATTTTCCAGC-3′	5′-CCAGAGGCGGTCACATCATA-3′
9	L1−DIAPH2	5′-TGCTGCTTCCTCTATCCCTG-3′	5′-GAGCTGAAACTTTGCTTGCAC-3′
10	L1−IGSF11	5′-CGCCTTGCAGTTTGATCTCA-3′	5′-GATGGTGACTGTTCCCTGGA-3′
11	L1−LINC00519	5′-CCAGTTTTCCCAGCACCATTTAT-3′	5′-AGGGAGAACAGCCAAGCC-3′
12	L1−ATP8B1	5′-CGCCTTGCAGTTTGATCTCA-3′	5′-CGTGGTACTTGCGATCGTTT-3′
13	L1−SVEP1	5′-TCAGCGAGATTCCGTGGG-3′	5′-ACTTCTACTCCACTTGCCGT-3′
12	L1−CD86	5′-AATCAGCGAGACTCCGTGG-3′	5′-ATCTTCAGAGGAGCAGCACC-3′
14	L1−SLC44A5	5′-TCGGAAAAGCGCAGTATTCG-3′	5′-CCCTTCTGGCCACAAAAGTG-3′
15	β-actin	5′- TGGCACCCAGCACAATGAA-3′	5′- CTAAGTCATAGTCCGCCTAGAAGCA-3′

Table S2. The list of antibodies in the research.

Immunohistochemistry.

Position	Antibody	Company	Dilution	Incubation
1	ATP8B1	Abcam	1:40	4°C overnight
2	CHKA	Proteintech	1:200	4°C overnight

Western blot.

Position	Antibody	Company	Dilution	Incubation
1	CHKA	Proteintech	1:1000	4°C overnight
2	AKT	CST	1:1000	4°C overnight
3	P−AKT	CST	1:1000	4°C overnight
4	ERK	CST	1:1000	4°C overnight
5	P−ERK	CST	1:1000	4°C overnight
6	PLA	CST	1:500	4°C overnight
7	PLC	CST	1:500	4°C overnight
8	PLD	CST	1:500	4°C overnight
9	GAPDH	CST	1:1000	4°C overnight

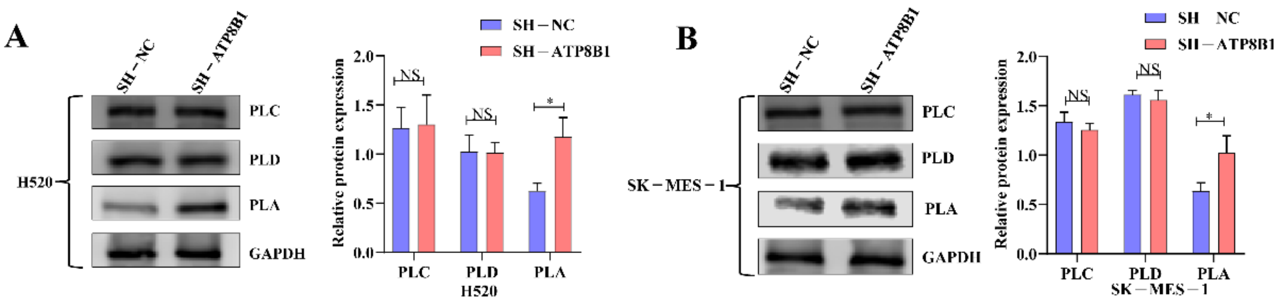

Figure S1. Phosphatidylcholine catabolism. (**A**) and (**B**) The protein expression levels of PLC and PLD did not change significantly in H520$^{SH-ATP8B1}$ and SK−MES−1$^{SH-ATP8B1}$ cells, while only PLA was up-regulated in H520$^{SH-ATP8B1}$ and SK−MES−1$^{SH-ATP8B1}$ cells. * $p < 0.05$.

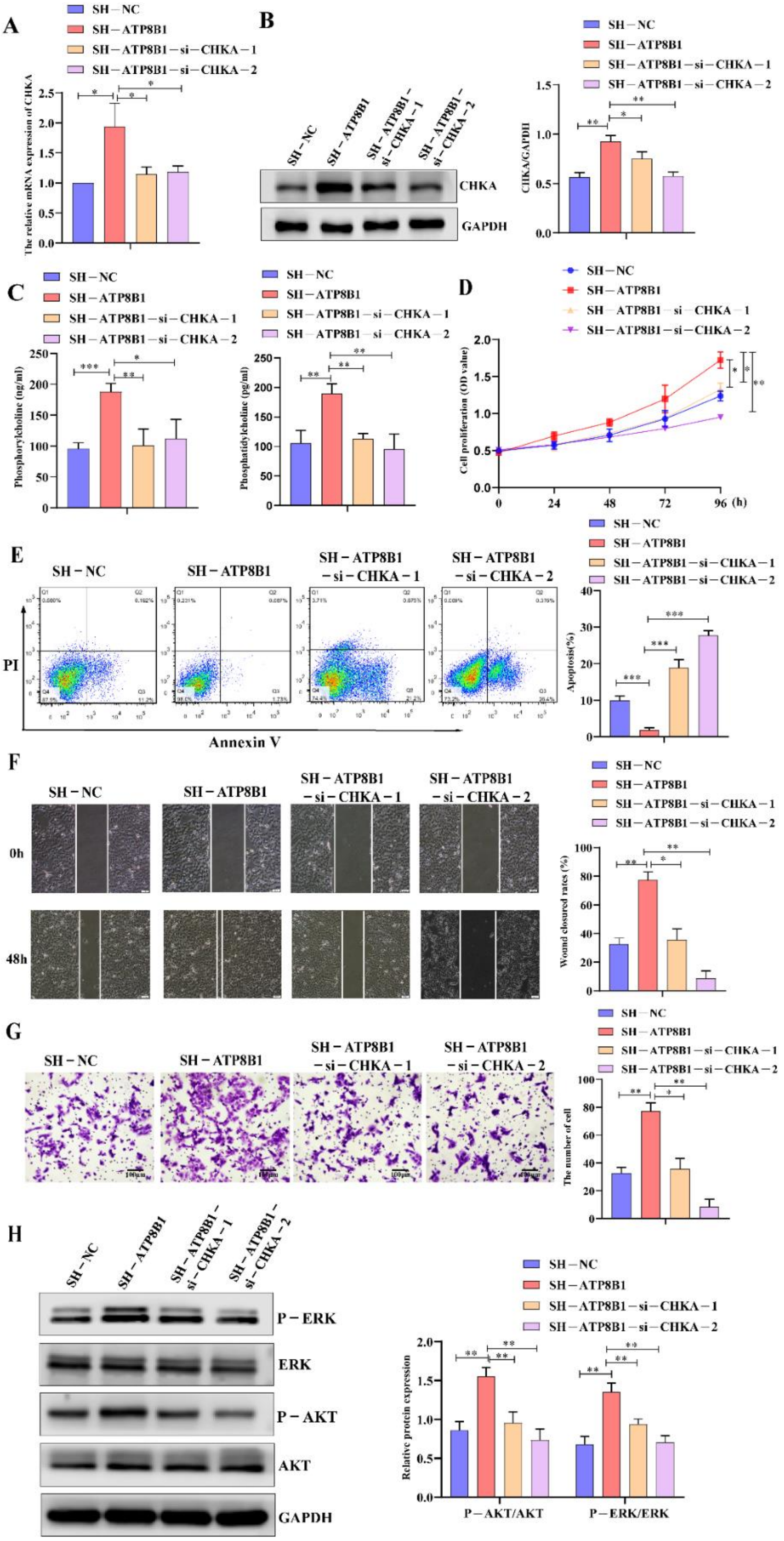

Figure S2. Interfering with CHKA expression in SH−ATP8B1 cell lines, inhibiting tumor progression**.** (**A**) Expression of CHKA in SK−MES−1 cells at mRNA level. All the RT−qPCR results shown for SK−MES−1$^{SH-ATP8B1}$ and SK−MES−1$^{SH-ATP8B1-si-CHKA}$ were relative mRNA expression values compared to SK−MES−1$^{SH-NCSH-NC}$ for normalization. (**B**) Expression of CHKA in SK−MES−1 cells at protein level. (**C**) The ELISA kit detects the level of phosphocholine in the cells. (**D**) Cell proliferation results. (**E**) Cell apoptosis results. (**F**) Representative images in wound healing assays. (**G**) Representative images in trans-well invasion assays. (H) Analysis of ERK and AKT activation by Western blot. * $p < 0.05$; ** $p < 0.01$; *** $p < 0.001$. Scale bar:100μm.

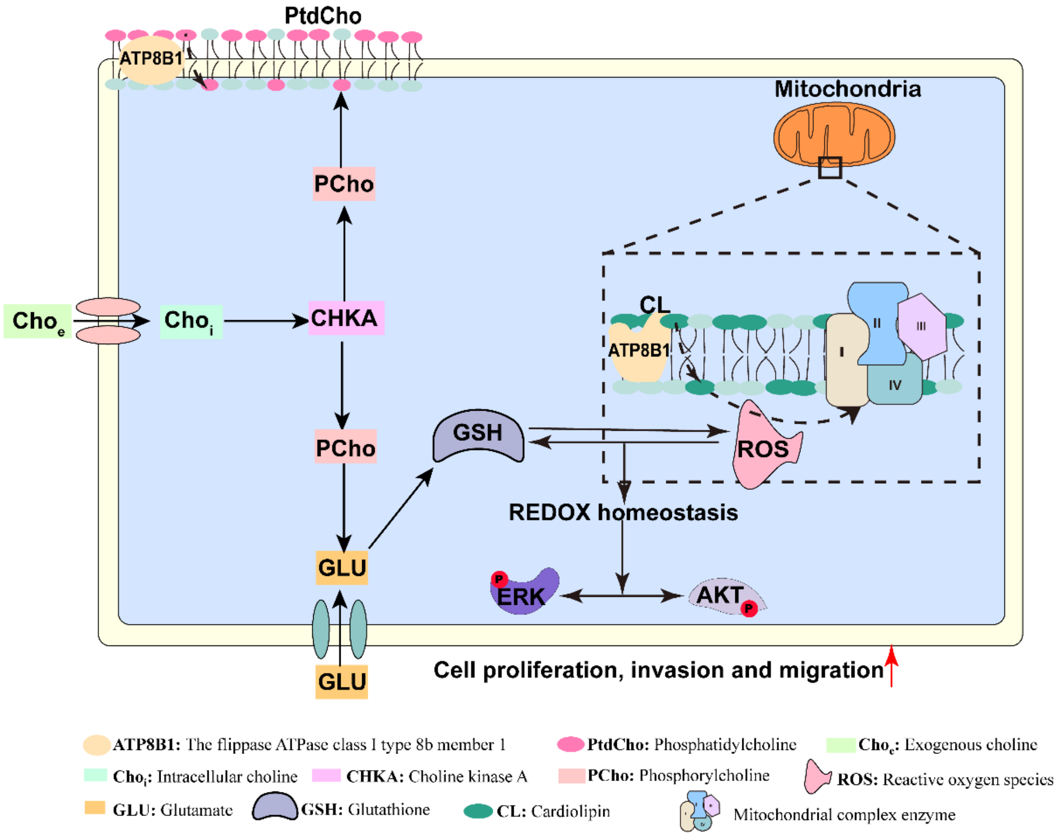

Figure S3. Model of ATP8B1 function. ATP8B1 encodes a cardiolipin transporter that mediates the flip of cardiolipin from the outside to the inside of the mitochondrial inner membrane. Cardiolipin was found to interact directly with a number of essential protein complexes, including respiratory chain complexes I through V. Defects in ATP8B1 can lead to cardiolipin remodeling, resulting in changes in cardiolipin homeostasis, resulting in decreased complex enzyme activity, and thus promoting ROS production. ATP8B1 mediates the internal turnover of phosphatidylcholine on the cell membrane. When ATP8B1 is defective, it activates the choline metabolism pathway. The activated CHKA promotes the uptake of glutamate and promotes the synthesis of GSH. ROS and GSH maintain the intracellular REDOX homeostasis, thereby activating the MAPK and PI3K/AKT pathways and promoting the malignant progression of tumors.

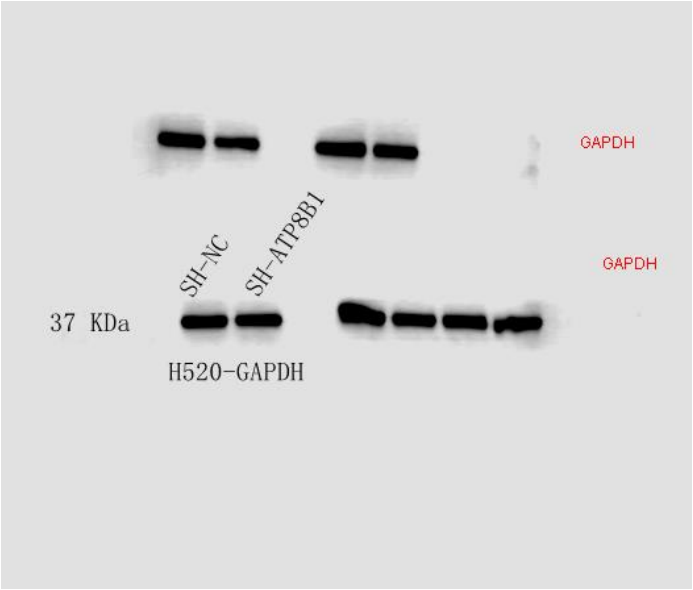

Figure 3D H520-GAPDH.

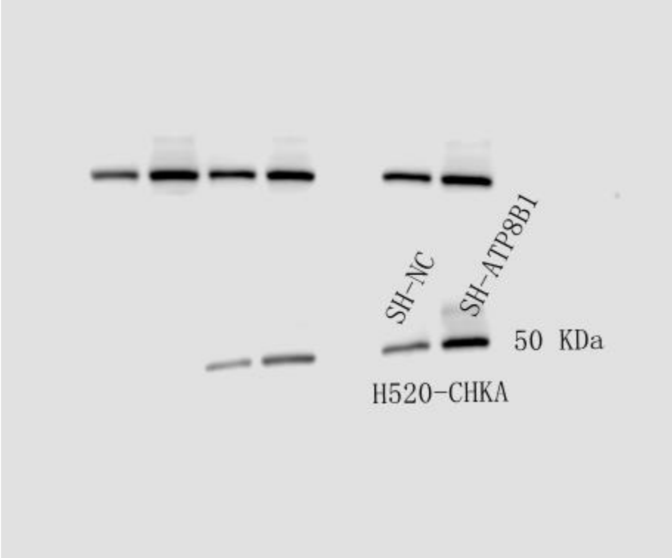

Figure 3D H520-CHKA.

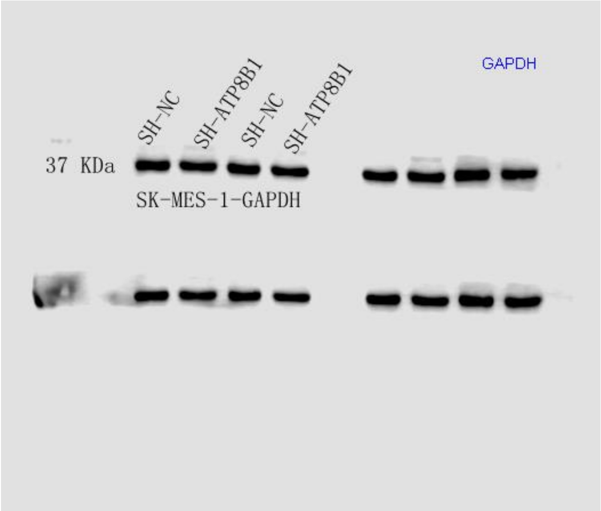

Figure 3SK—MES—1-GAPDH.

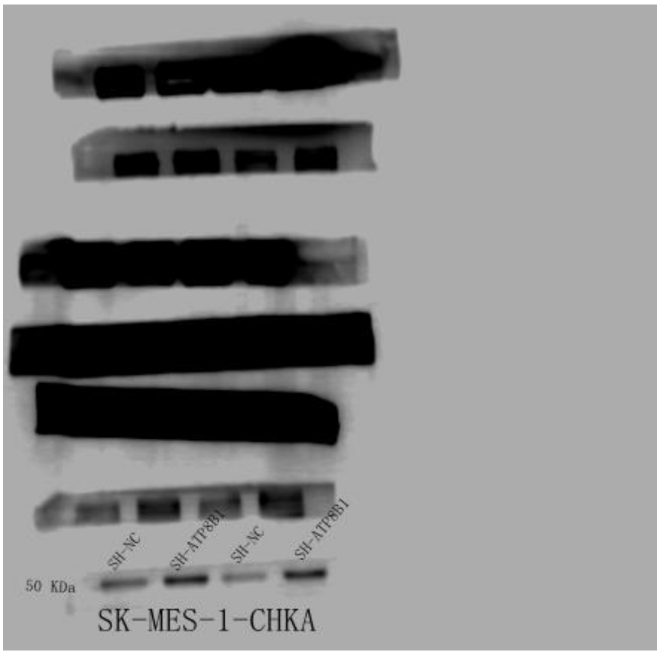

Figure 3D SK－MES－1-CHKA.

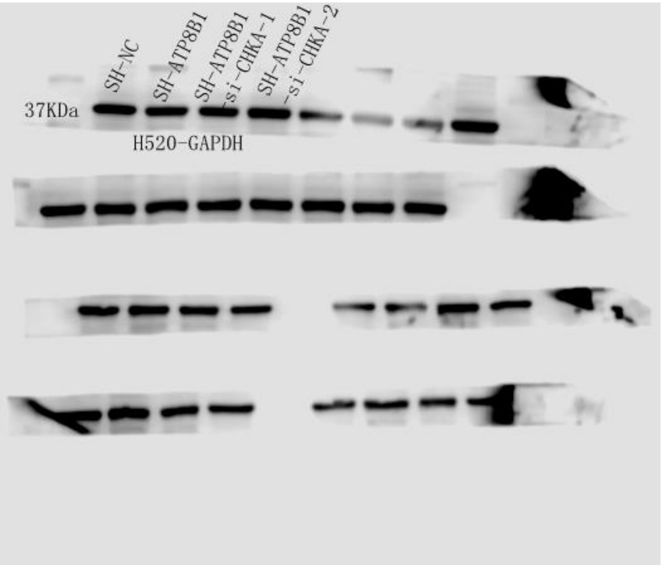

Figure 4B H520-GAPDH.

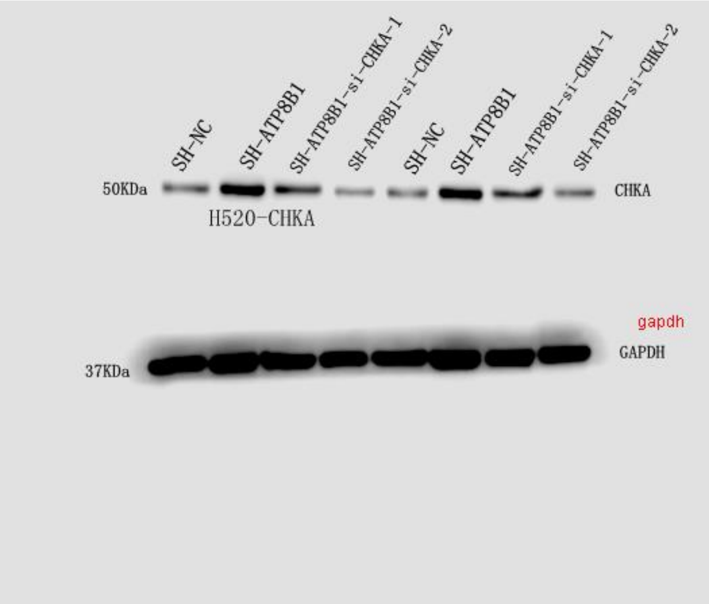

Figure 4B H520-CHKA.

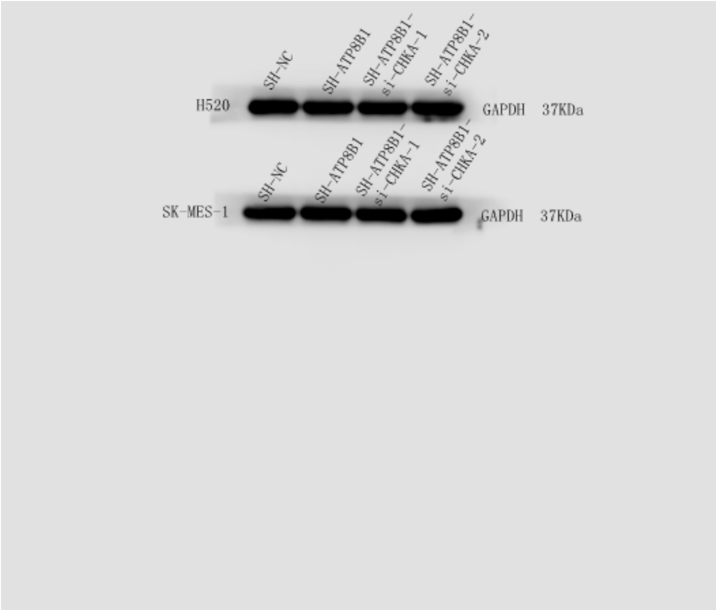

Figure 4H H520-GAPDH.

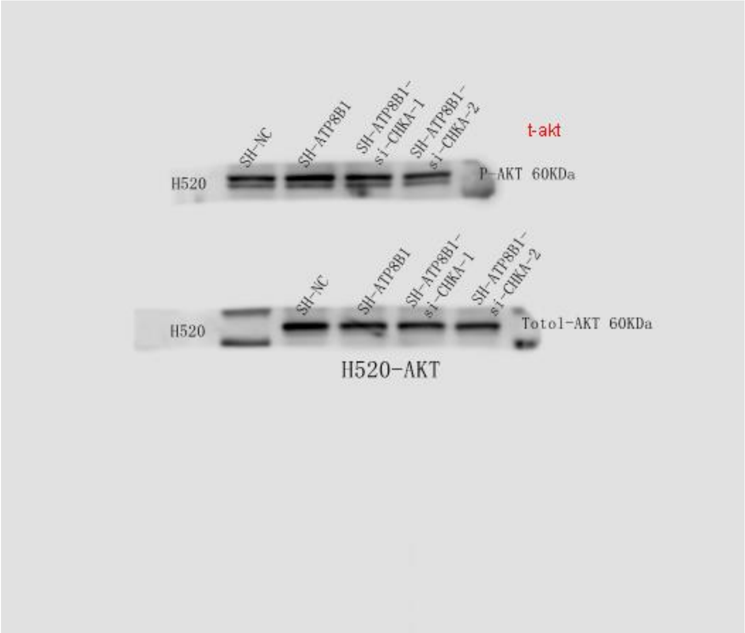

Figure 4H H520-AKT.

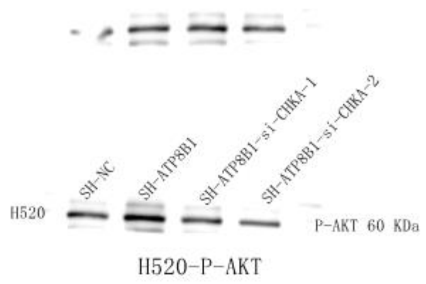

Figure 4H H520-P-AKT.

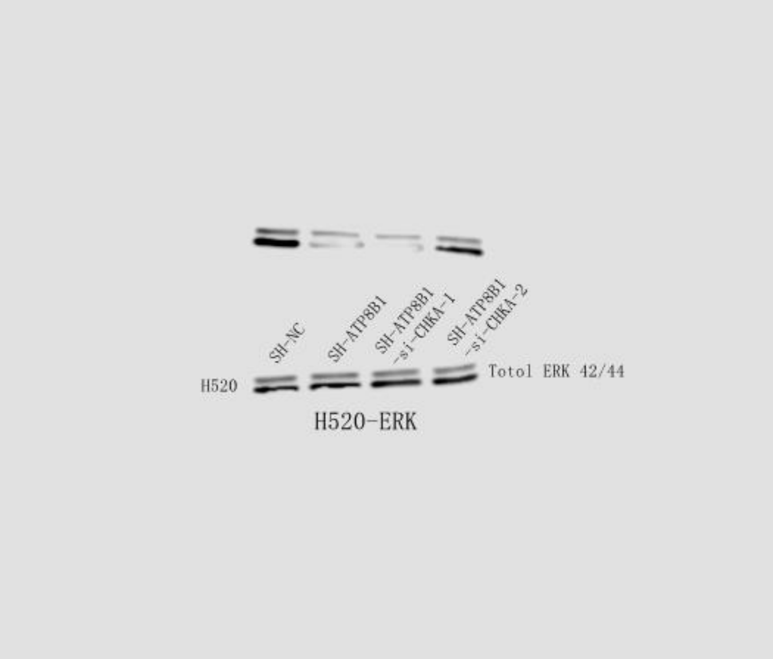

Figure 4H H520-ERK.

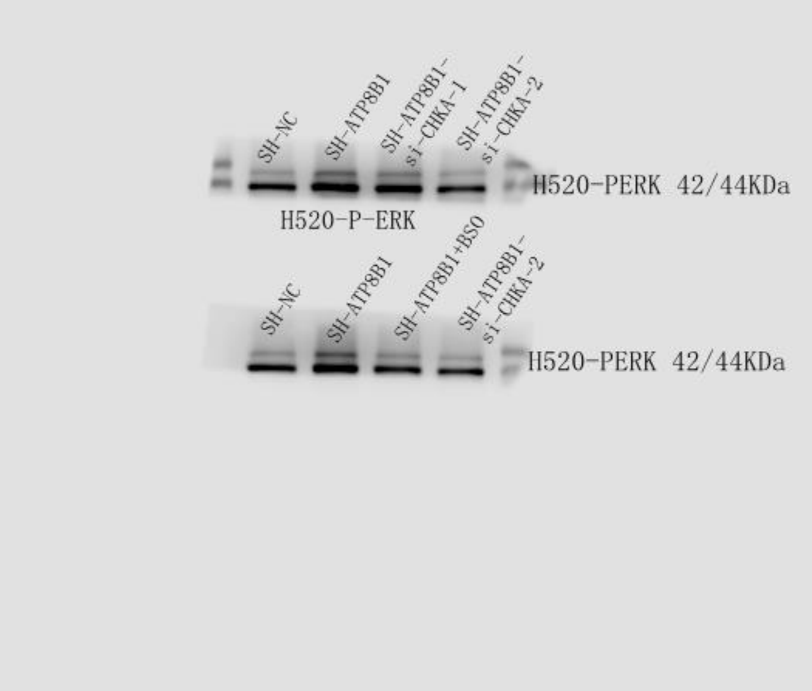

Figure 4H H520-P-ERK.

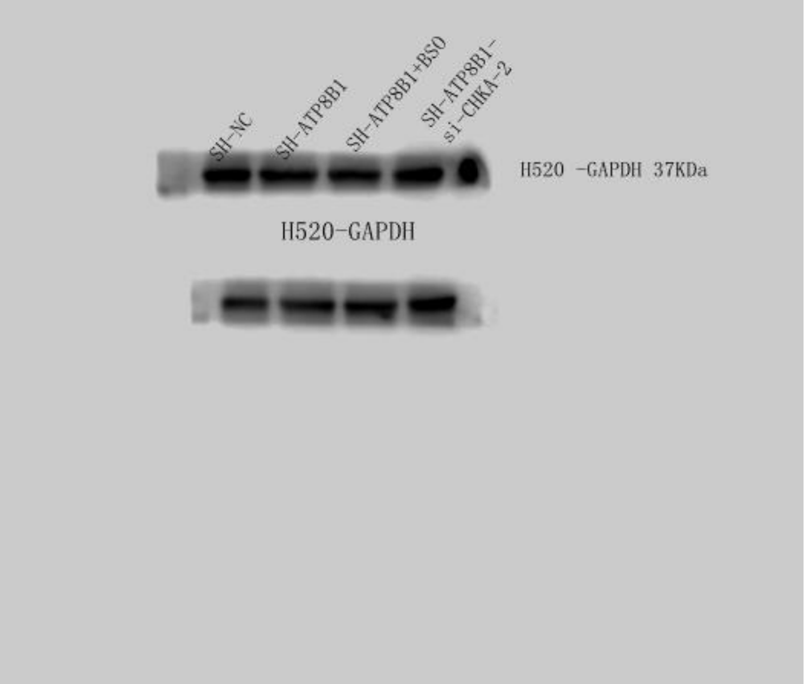

Figure 5I H520-GAPDH.

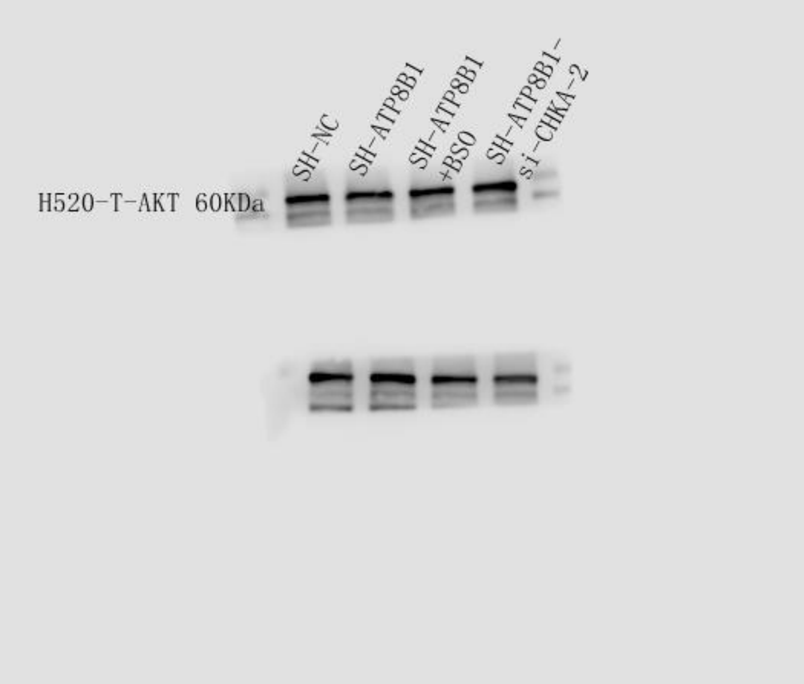

Figure 5I H520-AKT.

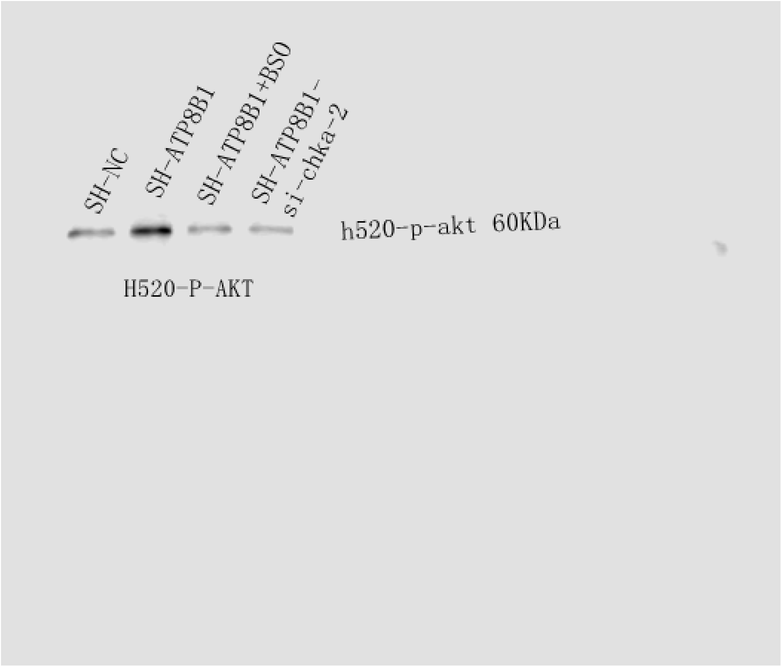

Figure 5I H520-P-AKT.

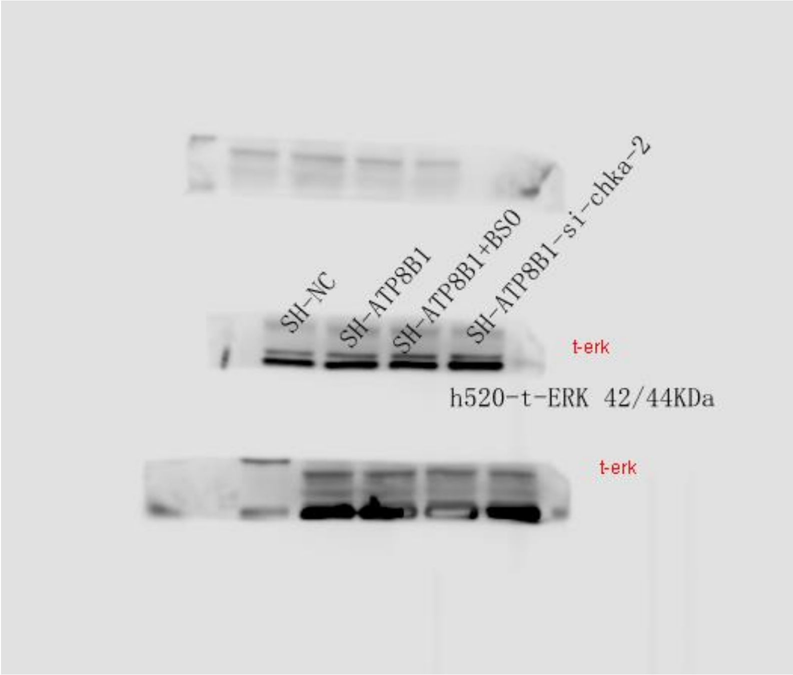

Figure 5I H520-ERK.

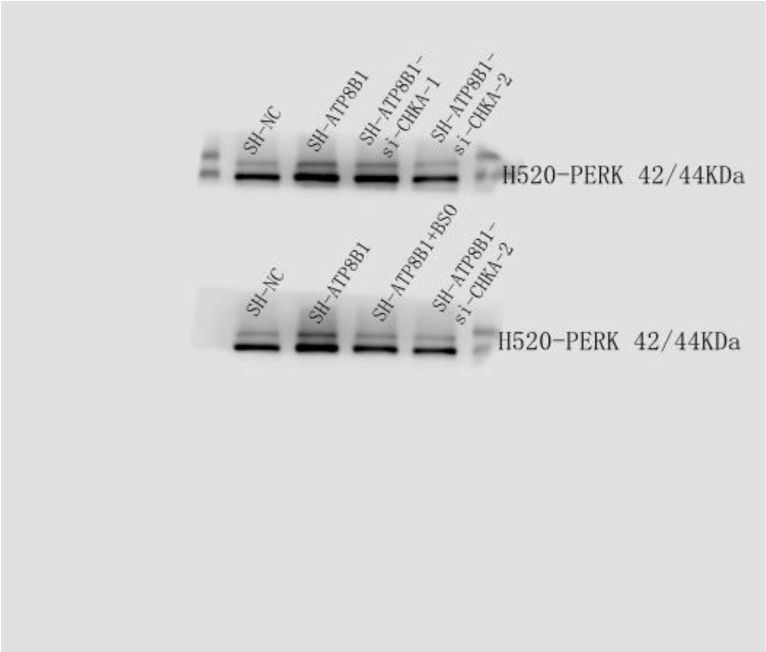

Figure 5I H520-P-ERK.

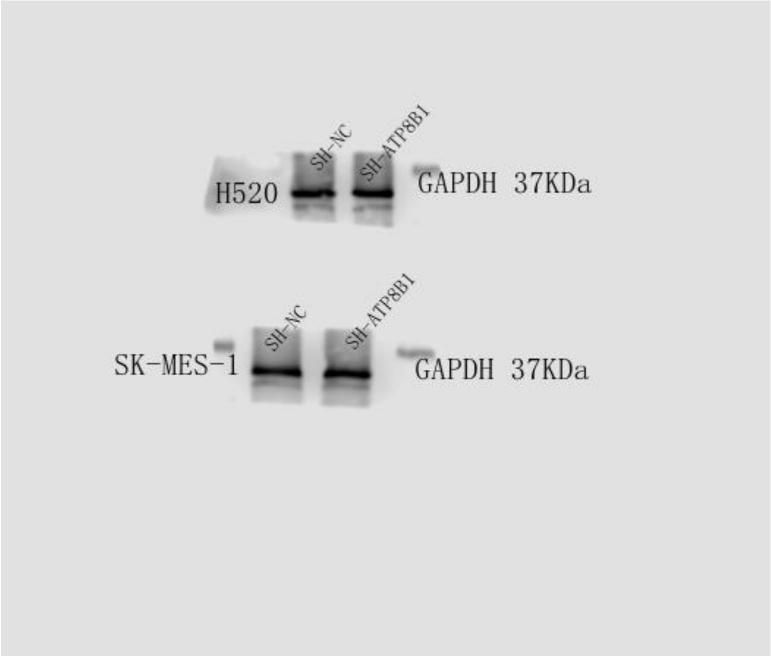

Figure S1A-B H520 and SK－MES－1-GAPDH.

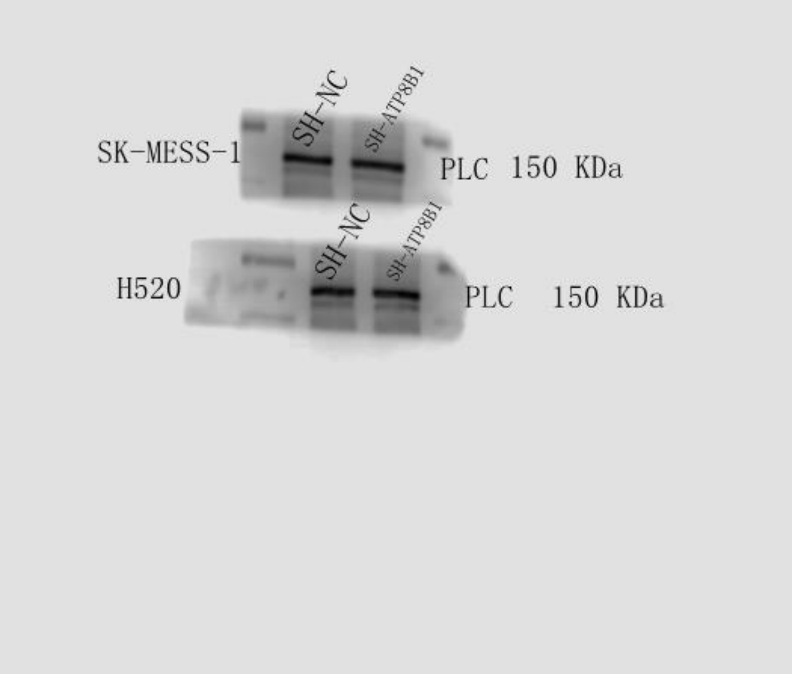

Figure S1A-B H520 and SK－MES－1-PLC.

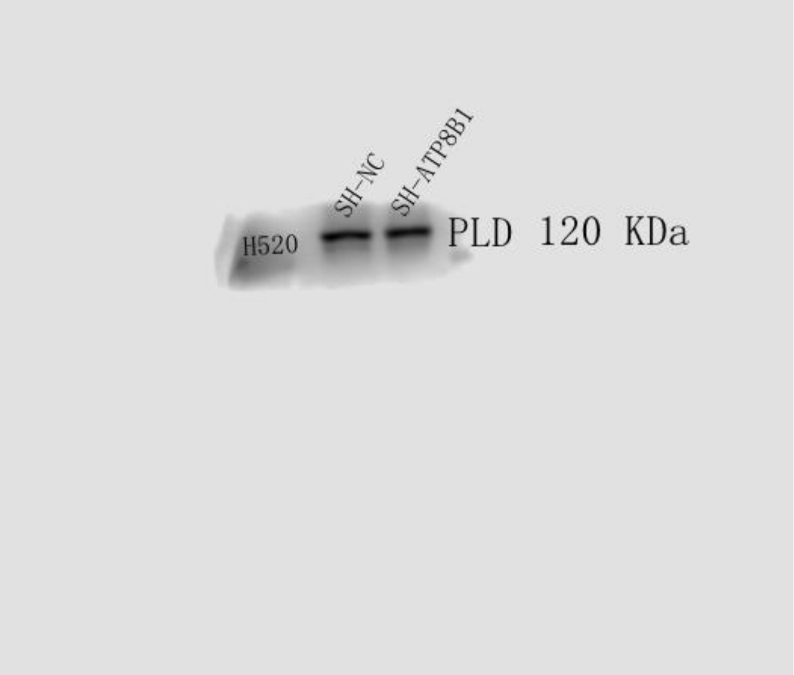

Figure S1A H520-PLD.

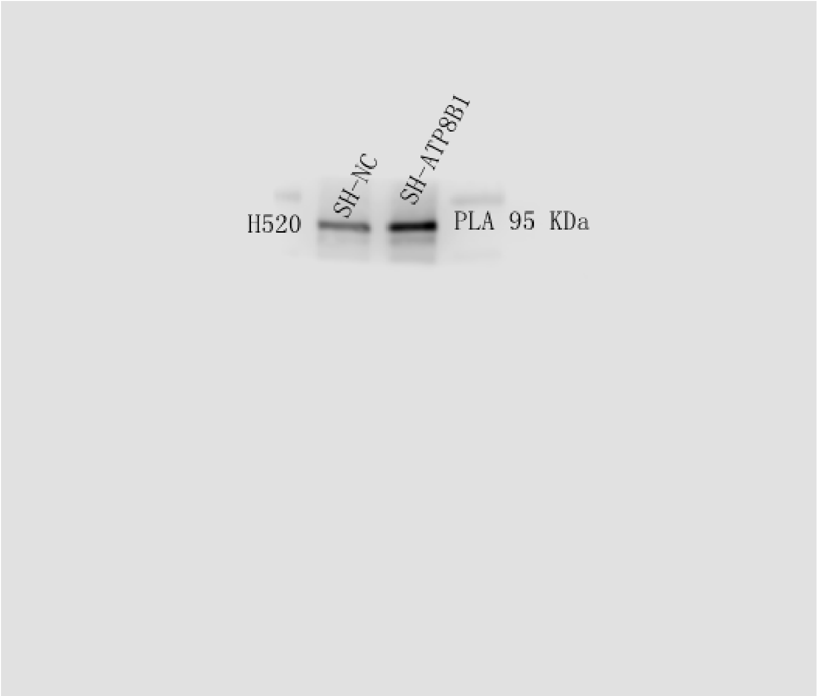

Figure S1A H520-PLA.

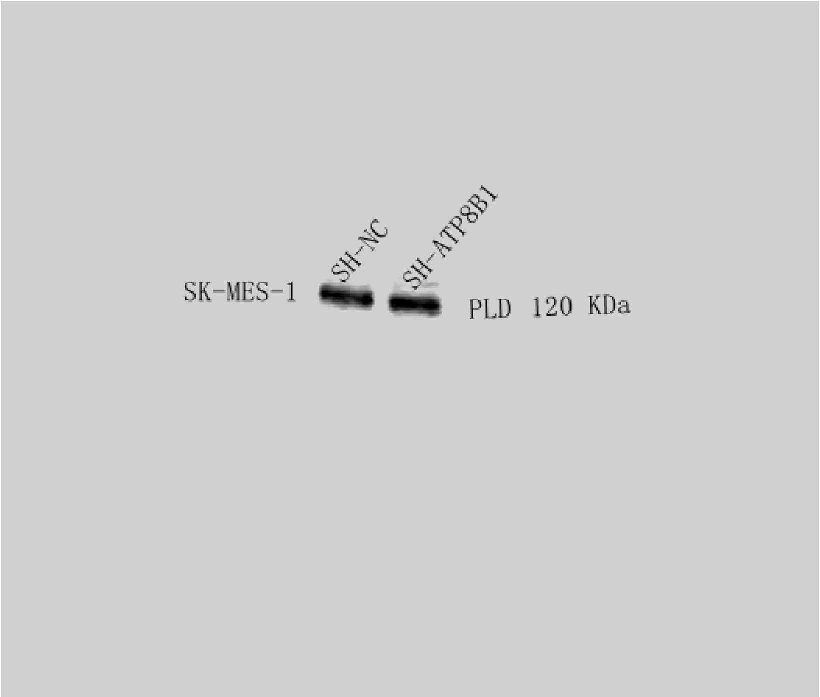

Figure S1B SK－MES－1-PLD.

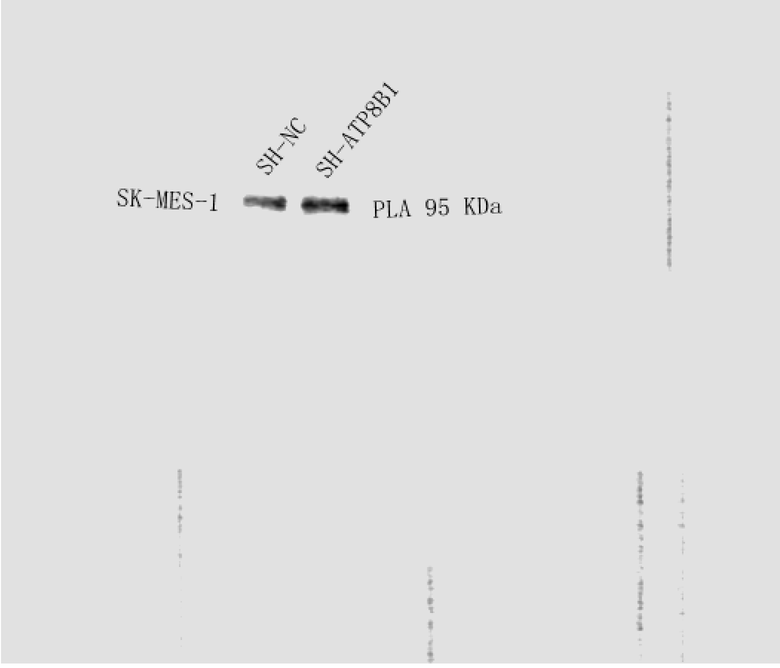

Figure S1B SK—MES—1-PLA.

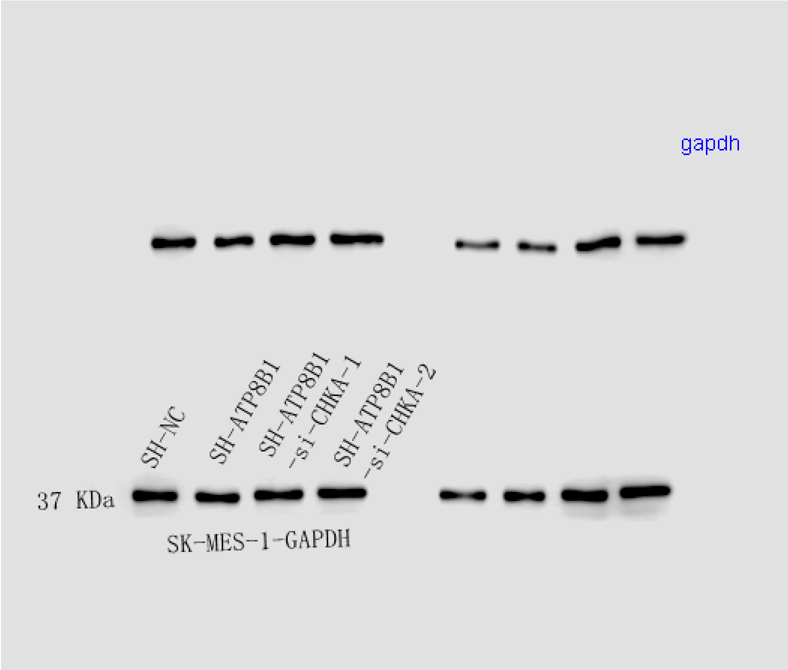

Figure S2B SK—MES—1-GAPDH.

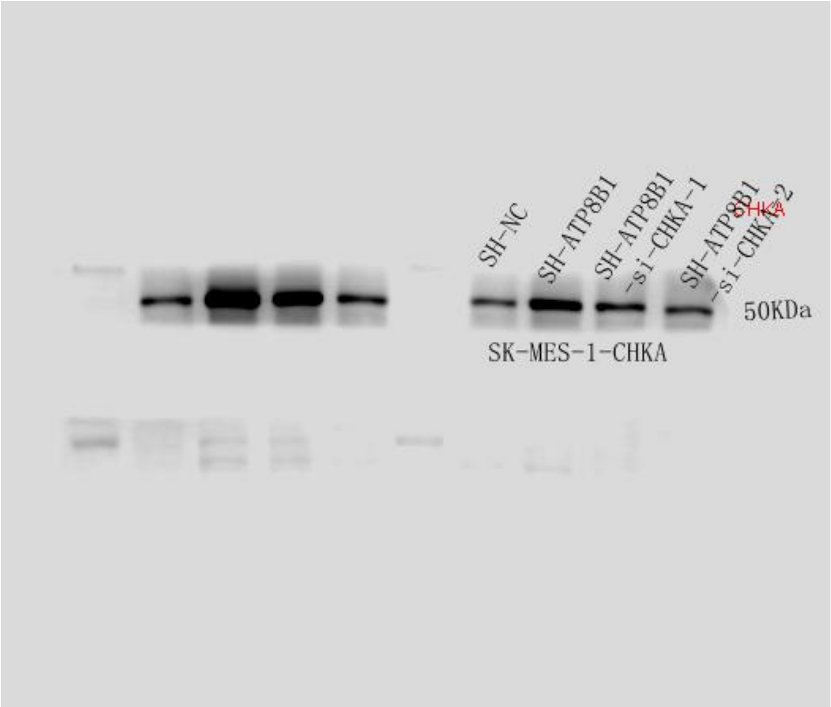

Figure S2B SK—MES—1-CHKA.

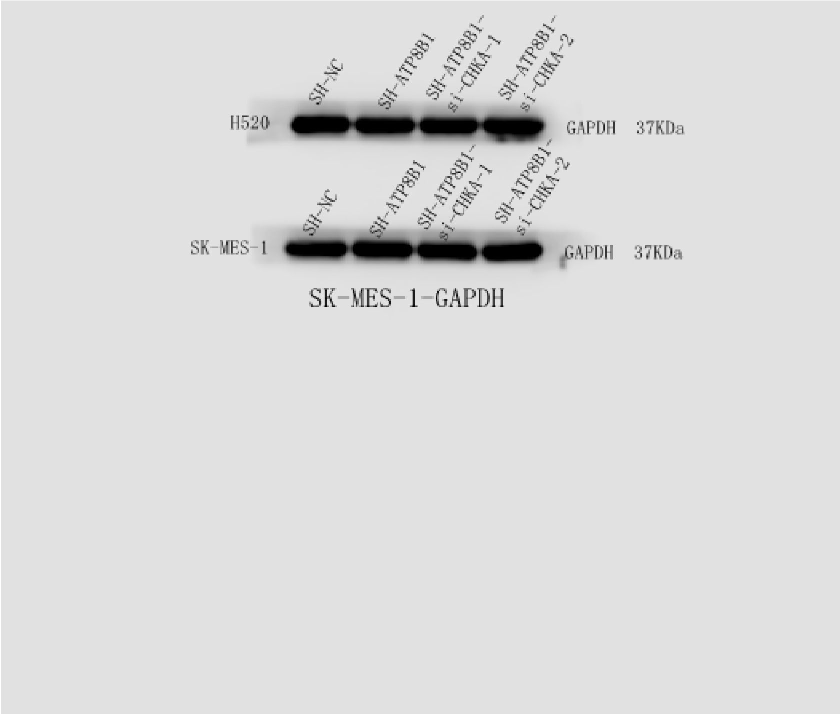

Figure S2H SK—MES—1-GAPDH.

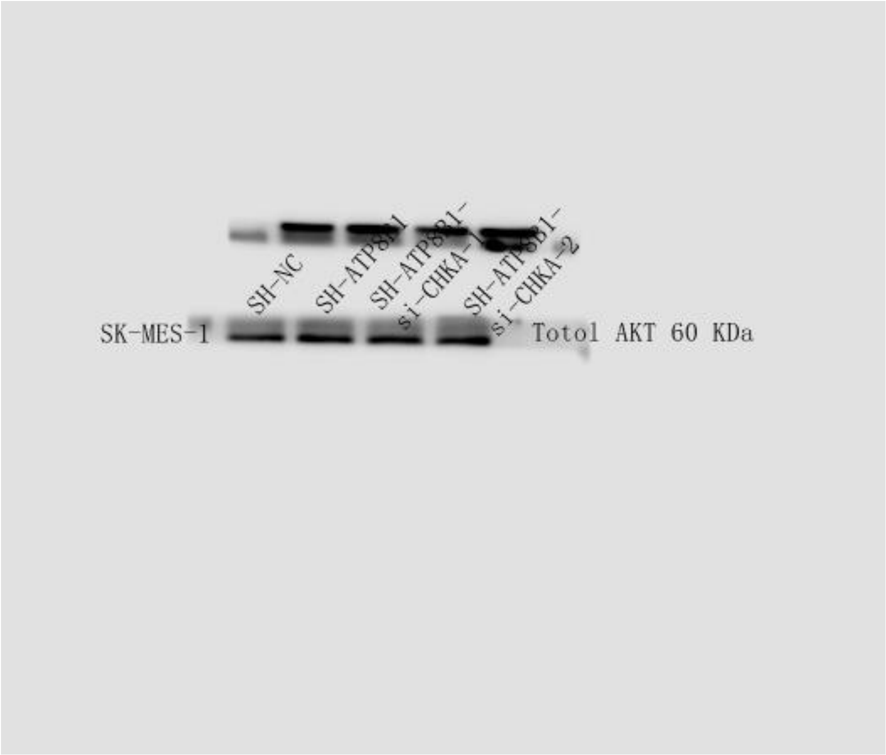

Figure S2H SK—MES—1-AKT.

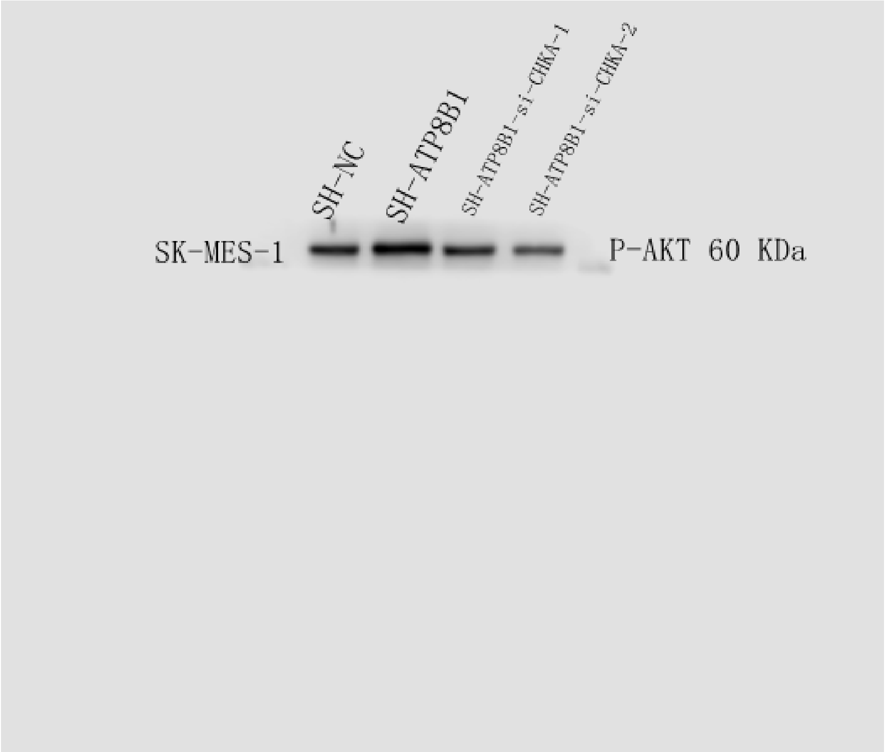

Figure S2H SK—MES—1-P-AKT.

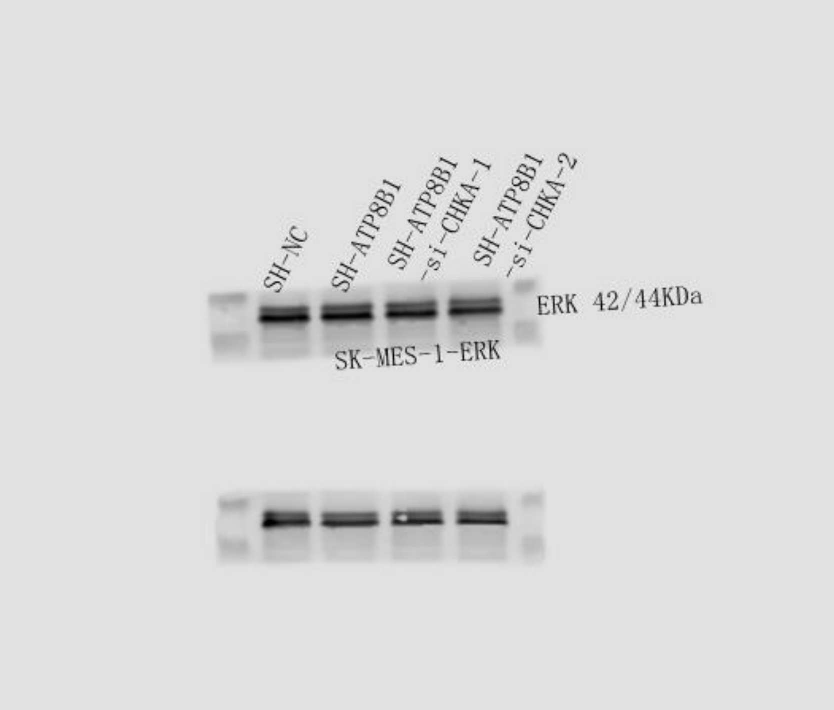

Figure S2H SK—MES—1-ERK.

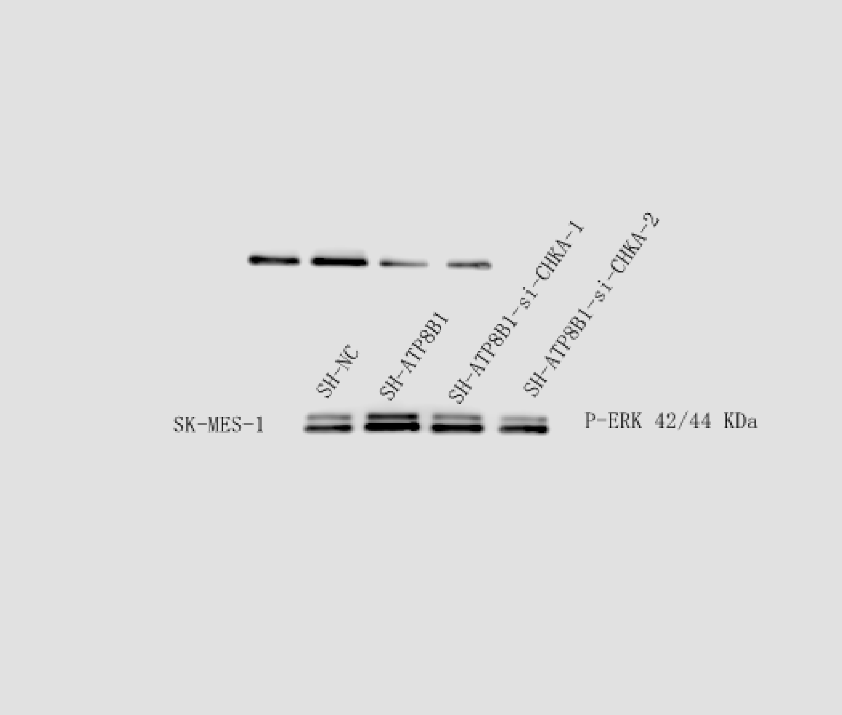

Figure S2H SK—MES—1-P-ERK.

Figure S4. Uncropeed western blot figures.
